# Efficacy of sequential letrozole and gonadotropin therapy for ovulation induction in women with polycystic ovary syndrome: a systematic review and meta-analysis

**DOI:** 10.1097/MS9.0000000000004506

**Published:** 2025-12-04

**Authors:** Waseem Sajjad, Muhammad Nabeel Saddique, Fatima Shahid, Anam Ijaz, Muhammad Atif Bashir, Muhammad Safiullah, Ursula Abu Nahla, Mohammad Rayyan Naseer, Malik Saad Hayat, Muhammad Hassan Zaman, Muhammad Usman, Basir Afzaal Gill

**Affiliations:** a Department of Obstetrics and Gynecology, King Edward Medical University, Lahore, Pakistan; b Faculty of Medicine, Hebron University, Hebron, Occupied Palestinian Territories; c Baim Institute for Clinical Research, Boston, Massachusetts, United States

**Keywords:** clomiphene citrate, gonadotropins, infertility, letrozole, polycystic ovary syndrome

## Abstract

**Background:**

Polycystic ovarian syndrome (PCOS) is a genetically diverse endocrine disorder affecting 5–20% of reproductive-aged women worldwide. It is a significant cause of hyperandrogenism, anovulatory infertility, menstrual dysfunction, and hirsutism. This meta-analysis assesses the effectiveness of sequential letrozole and gonadotropin therapy as compared to other ovulation induction regimens in women with PCOS.

**Methods:**

A comprehensive search was conducted through the PubMed, Embase, Cochrane, Scopus, and ClinicalTrials.gov databases for articles relevant from inception up to March 2024. Pooled outcome estimates were reported as odds ratios (ORs) and 95% confidence intervals (CIs) for dichotomous data, and as mean differences (MD) and 95% CI for continuous data. Statistical heterogeneity was assessed using *I*^2^ and *χ*^2^ statistics. All calculations were performed using RevMan 5.4.

**Results:**

This meta-analysis included 8 randomized controlled trials involving 932 anovulatory infertile women with PCOS diagnosed by the Rotterdam Criteria. We found a statistically significant increase in ovulation rates (OR 1.74; 95% CI 1.02–2.99; *P* = 0.04; *I*^2^ = 27%) in the treatment group compared to other ovulation induction regimens. There was no difference in pregnancy rates (OR 1.11; 95% CI 0.66–1.87; *P* = 0.69; *I*^2^ = 60%) and number of dominant follicles (MD 0.36; 95% CI −0.18 to 0.91; *P* = 0.19; *I*^2^ = 97%). The treatment group showed a statistically significant reduction in the size of dominant follicles (mm) (MD −0.27; 95% CI −0.95 to 0.41; *P* = 0.04; *I*^2^ = 88%) and an increase in endometrial thickness (mm) (MD 0.78; 95% CI 0.19–1.38; *P* = 0.010; *I*^2^ = 97%).

**Conclusion:**

The treatment group saw a significant rise in ovulation rates and endometrial thickness. Sequential letrozole and gonadotropin therapy (LE + G) may hold clinical significance, but further large-scale, high-quality studies are necessary to establish conclusive evidence.

## Introduction

Polycystic ovarian syndrome (PCOS) is a highly prevalent, genetically complex, and heterogeneous endocrine disorder in reproductive-aged women, affecting 5–20% worldwide^[1]^. It is a common cause of hyperandrogenism, anovulatory infertility, menstrual dysfunction, and hirsutism. It appears to be associated with an increased risk of metabolic abnormalities such as insulin resistance, hyperinsulinism, type 2 diabetes mellitus, dyslipidemia, cardiovascular diseases, and endometrial carcinoma^[2]^. PCOS accounts for more than 80% of women with anovulatory infertility. Ovulation induction therapy is a classical treatment for women with anovulatory PCOS who wish to conceive^[3]^.

The main treatment for PCOS involves ovulation induction, a way to stimulate growth until the maturation of a single follicle. For ovulation induction, several protocols are available, including pharmacological interventions such as clomiphene citrate (CC), aromatase inhibitors, gonadotropins, etc., and non-pharmacological modalities, including invasive laparoscopic ovarian surgery or ovarian drilling and intracytoplasmic sperm injection or *in vitro* fertilization. The non-pharmacological interventions are generally invasive, high cost, require hospitalization, need general anesthesia, less effectiveness, and pose a high risk of complications^[4]^.

CC, an estrogen receptor modulator, is a classical first-line pharmacological intervention for ovulation induction. It is a low-cost, orally administered drug with fewer side effects; 15% of women with PCOS are resistant to it. It may also induce ovarian hyperstimulation syndrome, inappropriate endometrial proliferation, and alterations in cervical mucus concentration^[4]^. CC is less effective than letrozole (LE) in terms of pregnancy rates and mono-follicular growth^[5]^. It is also associated with a high risk of miscarriages and prolonged depletion of receptors due to the anti-estrogenic effect of CC.

In recent years, LE, an aromatase inhibitor, in combination with gonadotropins, has shown promising results in women with PCOS, including CC-resistant PCOS patients. Gonadotropins, when given in combination with ovulation-inducing drugs, reduce the risk of complications while maintaining higher pregnancy rates. In clinical trials, LE plus gonadotropins sequential therapy has resulted in higher pregnancy rates and live births than any other intervention, including CC^[6]^. Moreover, it proved to be efficient in the case of CC-resistant PCOS patients due to its indirect effect on estrogen.

The main objective of using ovulation induction drugs is to achieve a higher pregnancy rate with fewer complications. Therefore, sequential LE plus gonadotropin therapy appeared to be an effective remedy. This systematic review aims to evaluate the effectiveness of sequential LE plus gonadotropin therapy in PCOS women, including those with CC-resistant PCOS. This study followed the transparency in the reporting of artificial intelligence (TITAN) guidelines 2025^[7]^.

## Methods

This systematic review and meta-analysis was conducted according to the Preferred Reporting Items for Systematic Review and Meta-Analyses (PRISMA) statement. This systematic review and meta-analysis was prospectively registered in the International Prospective Register of Systematic Reviews (PROSPERO).

### Search strategy and information sources

The PubMed, Embase, Cochrane, Scopus, and ClinicalTrials.gov databases were searched systematically by two independent authors (A.B. and M.N.) for articles relevant to the safety and effectiveness of sequential letrozole and gonadotropin (LE + G) therapy in infertile women with PCOS, using appropriate keywords, until March 2024. The line-by-line search strategy for each database is presented in the Supplemental Digital Content 1, available at: http://links.lww.com/MS9/B44.

### Study selection process

Studies were included in this systematic review if they met the following inclusion criteria: (1) Studies involving women diagnosed with PCOS based on the Rotterdam Criteria, requiring at least two of the following: oligo/anovulation, clinical or biochemical signs of hyperandrogenism, and polycystic ovaries on ultrasound; (2) Studies including patients with normal thyroid-stimulating hormone (TSH) and prolactin levels, and male partners with normozoospermia, as confirmed by semen analysis; (3) Studies involving women aged between 18 and 40 years; (4) Studies reporting patients with a history of infertility, defined as failure to conceive after at least one year of regular unprotected intercourse; (5) Studies assessing the efficacy of LE + G therapy for ovulation induction; (6) Only randomized controlled trials (RCTs) were considered to ensure high-quality, comparative evidence; (7) Studies must report key clinical outcomes, including ovulation rates, pregnancy rates, live birth rates, or other relevant reproductive metrics.

**HIGHLIGHTS**

- A systematic review and meta-analysis evaluating the efficacy of sequential letrozole and gonadotropin therapy for ovulation induction in women with polycystic ovary syndrome.
- Analysis of data from 8 randomized controlled trials, including 932 anovulatory infertile women, to assess the impact of the therapy on key reproductive outcomes.
- Significant improvement in ovulation rates in the treatment group, while no significant differences were observed in pregnancy rates, number of dominant follicles, or follicle size.
- Notable increase in endometrial thickness in the treatment group, suggesting a potential benefit for improving the uterine environment for implantation.
- Findings provide valuable insights into the effectiveness of sequential letrozole and gonadotropin therapy, particularly for women with clomiphene citrate-resistant polycystic ovarian syndrome.

The studies were excluded from the systematic review if: (1) Studies were excluded if they reported infertility due to conditions other than PCOS; (2) Studies involving patients with a history of ovarian surgery, endometriosis, or pelvic adhesions were not considered; (3) Studies involving patients with liver, kidney, or thyroid dysfunction were excluded to ensure a clear focus on PCOS-related infertility; (4) Studies involving women with congenital adrenal hyperplasia (CAH) or those who had undergone gynecological surgeries were excluded; (5) Women with autoimmune diseases, tubal obstruction, or allergic reactions to relevant ovulation induction medications were excluded from the review; (6) Studies utilizing cohort, case-control, cross-sectional designs, as well as letters to editors, conference abstracts, and case reports, were excluded to maintain methodological rigor and ensure high-quality evidence.

### Study selection process

The retrieved references were screened based on title, abstracts, and relevant outcomes and assessed for eligibility based on predefined inclusion and exclusion criteria by two reviewers (F.S. and M.H.Z.). A third independent reviewer (M.S.) was employed to resolve any discrepancies. The conference presentations and grey literature were not included in the literature search. The quality of evidence for each outcome was assessed using the GRADE approach with the GRADEpro GDT software^[8]^ (Supplemental Digital Content 1, available at: http://links.lww.com/MS9/B44).

### Data extraction and quality assessment

The data of the included studies were extracted by two independent reviewers (H.Z. and M.U.) regarding study characteristics (author, year, country, design), intervention and comparator regimens, duration of treatment, and primary and secondary outcomes. The outcomes of interest included ovulation rates, pregnancy rates, number of dominant follicles, the size of dominant follicles, serum estrogen levels, and endometrial thickness. The data was extracted into a standardized Excel sheet. A third independent reviewer (A.B.) was employed to resolve any discrepancy. The risk of bias of the included studies in five domains, i.e., selection bias, performance bias, detection bias, attrition bias, and reporting bias, was assessed using RoB 2.0 software by the Cochrane Collaboration.

### Statistical analysis

The primary endpoints of this meta-analysis were ovulation rates and pregnancy rates. The secondary endpoints were the number of dominant follicles, the size of dominant follicles (mm), endometrial thickness (mm), and serum E2 levels (pg/ml). Pooled outcome estimates for ovulation rates and pregnancy rates in the included studies were presented as percentages and odds ratio (OR) associated with 95% confidence intervals (CIs). The pooled outcome estimates of continuous outcomes like number of dominant follicles, size of dominant follicles (mm), endometrial thickness (mm), and serum E2 levels (pg/ml) were presented as mean difference (MD) associated with 95% CI. The interstudy heterogeneity was dealt with using a random effect model using the Mantel–Haenszel method for dichotomous outcomes and the Inverse Variance method for continuous outcomes. The heterogeneity among the studies was assessed by *I*^2^ statistics (*I*^2^ values < 50% = low heterogeneity; *I*^2^ values 50–75% = moderate heterogeneity; *I*^2^ values > 75% = significant heterogeneity). Sensitivity analyses were performed for larger studies. Funnel plots were not used for the assessment of the publication bias, as the recommended minimum of 10 studies per outcome was not reached^[9]^. All statistical calculations were performed using the meta-analysis software Review Manager 5.4.1 (The Cochrane Collaboration). A *P*-value of < 0.050 represented statistical significance.

## Results

### Study selection

**Figure 1.**
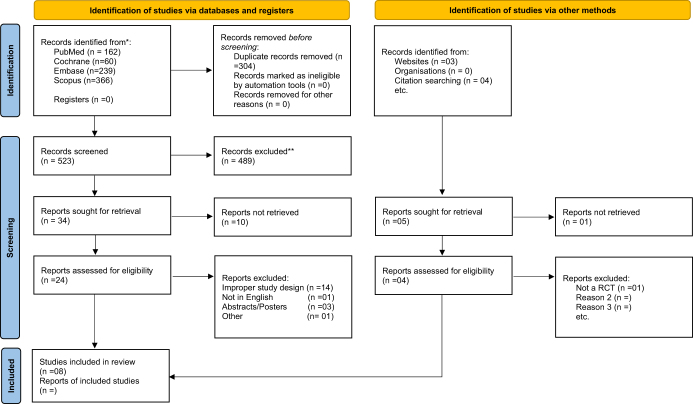
PRISMA flowchart outlining the study selection process for the systematic review and meta-analysis.

The refined search string yielded a total of 834 articles from all the information source databases. After excluding 304 redundant studies, the search resulted in 523 relevant studies. These retrieved studies underwent screening based on their titles and abstracts. Following the review of the full texts of potentially relevant studies, eight studies that met the selection criteria were included in this systematic review. The study selection process is illustrated in the PRISMA flowchart (Fig. 1).

## Risk of bias assessment

**Figure 2.**
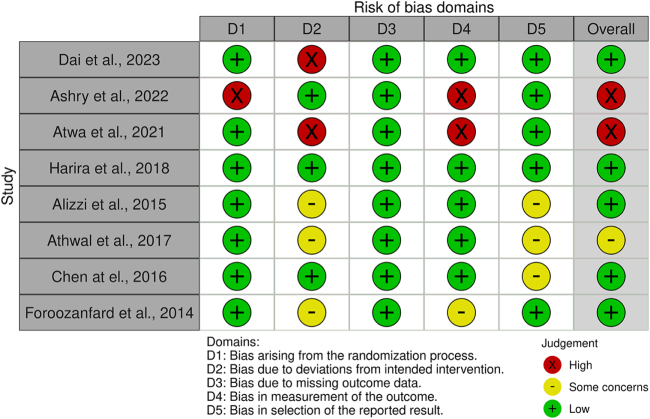
Traffic light plot of risk of bias: A visual representation of the authors’ judgments for each risk of bias domain in the included studies, using the Cochrane RoB 2.0 tool. Each domain is categorized as “low risk,” “some concerns,” or “high risk.”

**Figure 3.**
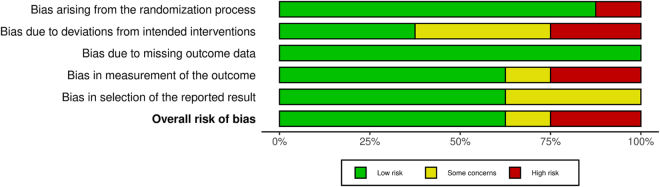
Risk of bias summary graph: Proportion of studies evaluated across five different bias domains (randomization process, deviations from intended interventions, missing outcome data, measurement of outcomes, and selection of reported results) with corresponding bias levels.

The risk of bias was assessed using RoB 2.0 by the Cochrane Collaboration^[10]^. The overall risk was considered “low” due to missing outcome data and bias arising from the randomization process. There were “some concerns” regarding bias in the selection of reported outcomes and a somewhat “high” risk of bias in deviations from intended interventions and in the measurement of outcomes in all the included studies (Figs 2 and 3).

### Baseline characteristics and patients’ demographics

This systematic review and meta-analysis comprised eight studies published from 2014 to 2023. All the included studies were RCTs. The number of participants ranged from 27 to 106 patients in both groups. A total of 956 patients were included in this systematic review and meta-analysis. The overall mean age for the treatment group was approximately 27.16 years. The treatment protocols included letrozole at 2.5–5 mg daily for 5 consecutive days, starting on any day between the second and seventh day of the menstrual cycle. It was often combined with gonadotropins such as FSH (75 IU) or highly purified HMG, depending on patient factors like age, BMI, and medical history. Ovulation was triggered with an intramuscular injection of 10 000 IU HCG when the leading follicle reached a size of 17–18 mm. Some protocols included the use of ethinylestradiol and cyproterone acetate as pretreatment or insulin sensitizers like metformin in patients with insulin resistance. Luteal phase support (LPS) with progesterone was initiated in some cases after HCG triggering to enhance pregnancy outcomes (Table 1).

**Table 1:** Baseline characteristics and demographic details of patients in the included studies

Study ID	Country	Study setting	Study design	No. of patients (treatment/control)	Inclusion criteria	Exclusion criteria	Mean age (treatment/control) ± SD	Intervention in treatment arm	Intervention in control arm	Trial funding	Trial registration
Ashry *et al* 2022	Egypt	Multicenter	RCT	90 (45/45)	Women with infertility and PCOS (Rotterdam criteria)	Infertility without meeting the Rotterdam criteria	(24.2 ± 3.1)/(26.0 ± 3.5)	Letrozole 2.5 mg twice daily (second to fifth days), gonadotropins from 7th day, HCG 10 000 IU when follicle reaches 17–18 mm	Letrozole 2.5 mg twice daily (second to fifth days), HCG 10 000 IU when follicle reaches 17–18 mm	Not given	Not given
					Age < 35 years	Age > 35 years					
					Normal TSH and prolactin levels	Abnormal TSH or prolactin levels					
					Day 3 FSH < 12 IU/ml	Abnormal semen analysis					
					Normal hysterosalpingography (HSG)	Elevated day 3 FSH levels					
					Male partner with abnormal semen analysis						
Atwa *et al* 2021	Egypt	Single center	RCT	54 (27/27)	Women aged 18–40 years	Thyroid disorders, diabetes mellitus, hyperprolactinemia, or Cushing syndrome	(27.8 ± 2.3)/(25.7 ± 3.4)	Letrozole 2.5 mg twice daily (from day 2 for 5 days), FSH 75 IU daily from day 3, HCG 10 000 IU when follicle ≥18 mm	Trans-vaginal needle ovarian puncture, Letrozole 2.5 mg twice daily (from day 2 for 5 days), FSH 75 IU daily from day 3, HCG 10 000 IU when follicle ≥18 mm	Self-funded research	Not given
					Diagnosed with PCOS (Rotterdam ESHRE/ASRM criteria)						
						History of ovarian drilling					
					Clomiphene citrate resistant						
						Abnormal hysterosalpingogram					
					Normal TSH and prolactin levels						
						Ovarian cyst on day 2 of the cycle					
					No ovulation induction medications in the prior 2 months						
						Abnormal semen parameters					
Dai *et al* 2023	China	Single center	RCT	174 (87/87)	Women with PCOS meeting the 2003 Rotterdam criteria	Allergy to study drugs	(27.00 ± 2.97)/(27.11 ± 3.20)	Ethinylestradiol and cyproterone acetate tablets (1–3 months), Letrozole 2.5 mg daily (cycle days 3–7), HMG 75 IU daily (cycle days 8–10), HCG 10 000 IU when follicle ≥18 mm	Ethinylestradiol and cyproterone acetate tablets (1–3 months), Letrozole 2.5 mg daily (cycle days 3–7), HCG 10 000 IU when follicle ≥18 mm	Not given	Registered as Chinese Clinical Trial Registration ChiCTR1900022971 (www.chictr.org.cn; registration date 5 May 2019)
						Inability to cooperate with protocol					
					Age 18–40 years						
					History of unprotected intercourse for >1 year	Severe cardiovascular disease					
						Severe hepatic or renal dysfunction					
					Normal uterine morphology and at least one patent Fallopian tube on hysterosalpingography						
						Pregnancy or lactation					
					Normal semen analysis of the spouse	History of abnormal uterus or uterine cavity disease					
					Normal organ function without endocrine disorders	Previous exposure to teratogens or radiation					
Alizzi *et al* 2018	Iraq		Prospective RCT	149 (25/124)			(28.0 ± 2.3) in the treatment arm	Letrozole 5 mg (days 2–3 of cycle for 5 days), FSH added if no response, hCG triggered when follicle ≥17 mm, LPS for 14 days in some	Letrozole 5 mg (days 2–3 of cycle for 5 days), FSH added if no response, hCG triggered when follicle ≥17 mm, LPS for 14 days in some		
Athwal *et al* 2017	India		Pilot Study (RCT)	124 (65/59)			(27.3 ± 1.9)/(26.9 ± 2.1)	Letrozole 5 mg/day (days 3–7 of menstrual cycle), FSH 75 IU daily (days 7–9), hCG 10 000 IU triggered when follicle ≥18 mm	Clomiphene citrate 100 mg/day (days 3–7 of menstrual cycle), FSH 75 IU daily (days 7–9), hCG 10 000 IU triggered when follicle ≥18 mm		
Chen *et al* 2016	China	Single center	RCT	156 (52/104)	Age 23–38 years (mean 26.9 ± 5.1)	Infertility due to non-PCOS ovulatory disorder or other causes	(27.7 ± 5.2)/(26.4 ± 4.2)	Letrozole 2.5–5.0 mg/day (days 3–5), HMG 75 IU every other day for 5 days	52 patients received Letrozole 2.5–5.0 mg/day (days 3–5), 52 patients received Clomiphene citrate 50–100 mg/day (days 3–5)	None	Not given
					Infertility duration: 1–12 years (mean 3.2 ± 1.0)						
						History of ovarian surgery, endometriosis, or pelvic adhesions					
					Diagnosed with PCOS per 2003 Rotterdam criteria						
						Liver, kidney, or thyroid dysfunction					
					At least one patent fallopian tube confirmed by salpingography or hydrotubation						
						Did not complete treatment per protocol or withdrew midway					
					Normal semen analysis in the male partner						
Foroozanfard *et al* 2014	Iran	Single center	RCT	198 (50/148)	Age between 20 and 35 years	Infertility without meeting the Rotterdam criteria	(25.8 ± 3.30)/(28.43 ± 4.43)	Letrozole 5 mg/day (days 3–7), HMG 150 IU (days 5–10), hCG 5000 IU when follicle ≥18 mm, followed by LPS (Progesterone suppositories for 14 days)	Clomiphene citrate 100 mg/day (days 3–7), HMG 150 IU (days 5–10), hCG 5000 IU when follicle ≥18 mm, followed by LPS (Progesterone suppositories for 14 days)	Funded by grant from Kashan Medical University, Kashan, Iran	Not given
					Married	Age > 35 years					
					No non-classical adrenal hyperplasia	Abnormal TSH or prolactin levels					
					No thyroid disorders or hyperprolactinemia						
					Iranian nationality						
					Effective speaking and listening skills						
					No male factor infertility	Abnormal semen analysis					
					Normal uterine cavity and at least one patent fallopian tube (confirmed by HSG, laparoscopy, or hysteroscopy)						
					Diagnosed with PCOS based on Rotterdam criteria						
Harira *et al* 2018	Egypt	Single center	RCT	212 (106/106)	CC-resistant PCOS (anovulation despite 2–3 cycles of 100–150 mg CC)	Infertility factors other than anovulatory PCOS (e.g., hypothalamic amenorrhea)	(26.5 ± 3.5)/(25.9 ± 2.95)	Letrozole 5 mg (day 3 for 5 days), Urofollitropin FSH on alternate days starting from day 7, hCG 5000 IU triggered.	Letrozole 5 mg + Clomiphene citrate 100 mg (for 5 days from day 3 of menstruation)	Not given	Not given
					Age ≤ 35 years						
					Patent both tubes confirmed by HSG or laparoscopy						
					No history of pelvic surgery or pelvic inflammatory disease						
					No history of exogenous gonadotropin treatment						
					No history of laparoscopic ovarian drilling or cystectomy						
					Normal semen analysis of partner						
						Uterine pathology (leiomyoma, adenomyosis, or congenital uterine malformation)					
						Hypersensitivity or contraindications to letrozole					
Hosseini-Najarkolaei *et al*, 2020	Iran	Single center	RCT	116 (57/59)	PCOS diagnosis based on Rotterdam criteria (≥2 of 3):	Age > 35 years	30.12 ± 0.33/ 29.4 ± 0.42	**LE + G**	**Artificial endometrial preparation**	Did not receive a funding grant	Registered in the Iranian Registry of Clinical Trials (www.irct.ir;IRCT20090526001952N12)
								Letrozole 5 mg orally twice daily for 5 days from day 3 of cycle			
						Uterine factors					
						Severe male factor infertility					
									Days 2–3: Confirm pituitary desensitization with ultrasound and E2 check.		
					Oligo-ovulation or anovulation						
						Severe endometriosis					
					Clinical or biochemical hyperandrogenism						
								HCG 10 000 IU intramuscularly (75–150 units) from day 5 to 9 to trigger ovulation			
						Immunologic disorders					
									If <5 mm thickness, start 4 mg estradiol valerate (increased to 6 mg). After 7 days, if ≥7 mm, continue same estradiol, add 50 mg progesterone for 2–3 days, and determine embryo transfer day. If not, increase to 8 mg/day until proper thickness endometrial thickness		
					PCO morphology on ultrasound (≥12 follicles or ovary volume ≥10 cm^3^)						
						Candidates for preimplantation genetic detection					
						History of recurrent miscarriage or repeated implantation failure					
						Basal FSH > 10 IU/ml					
						BMI ≥ 30 kg/m^2^					

RCT, randomized controlled trial; LE, letrozole; HCG, human chorionic gonadotropin; FSH, follicle-stimulating hormone; HMG, human menopausal gonadotropin; LPS, luteal phase support; CC, clomiphene citrate.

## Outcomes (Table 2)

### Ovulation rates

**Figure 4.**
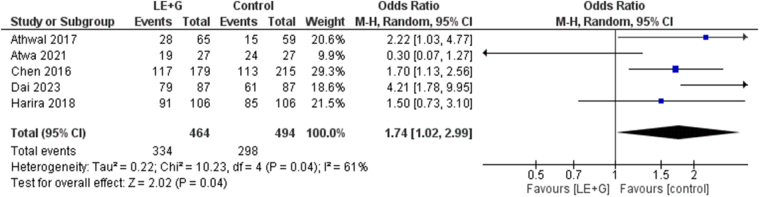
Forest plot showing the comparative analysis of ovulation rates between the sequential letrozole and gonadotropins therapy and control groups.

The comparative analysis showed a significant increase in ovulation rates (OR 1.74; 95% CI 1.02–2.99; *P* = 0.04; *I*^2^ = 61%) in the treatment group (Fig. 4). When the sensitivity analysis was performed to deal with the heterogeneity, the exclusion of *Atwa et al* (2021) resulted in significantly higher rates of ovulation in the control group (OR 2.02; 95% CI 1.38–2.95; *P* = 0.0003, *I*^2^ = 27%).

**Table 2:** Summary table of outcomes of the included studies

Publication	Intervention	Population	Outcomes					
			Ovulation rate	Pregnancy rate	No. of dominant follicles	Size of dominant follicles (mm)	Endometrial thickness (mm)	Serum E2 levels (pg/ml)
Ashry *et al*, 2022	LE + HCG + G	45	NR	14/45 (31.1%)	2.4 ± 0.8	NR	7.8 ± 1.8	981.1 ± 126.7
	LE + HCG	45	NR	14/45 (31.1%)	2.0 ± 0.7	NR	7.6 ± 1.2	946.7 ± 152.8
Atwa *et al*, 2021	LE + FSH + HCG	27	19/27 (66.6%)	4/27 (14.8%)	0.86 ± 0.72	17.92 ± 2.2	NR	NR
	Trans-vaginal needle ovarian puncture before ovulation induction by LE + G	27	24/27 (89%)	9/27 (33.3%)	1.1 ± 0.68	19.89 ± 2.92	NR	NR
Dai *et al*, 2023	LE + HCG + HMG	87	79/87 (90.8%)	Conception 29/87 (33.3%); clinical pregnancy 24/87 (27.6%)	NR	NR	9 (7–10)	957 (638–1360)
	LE + HCG	87	61/87 (70.1%)	Conception 15/87 (17.2%); clinical pregnancy 12/87 (13.8%)	NR	NR	8 (7–10)	916 (591–1198)
Alizzi *et al*, 2018	LE + Recombinant G (FSH)	25	NR	7/25 (28.0%)	20/25 (80.0%)	19.32 ± 1.02	NR	8.03 ± 0.40
	LE + G with LPS	25	NR	11/25 (44.0%)	22/25 (88.0%)	19.17 ± 1.21	NR	7.92 ± 0.21
	LE only	49	NR	15/49 (30.6%)	48/49 (98.0%)	18.59 ± 0.70	NR	7.68 ± 0.45
	LE with LPS	50	NR	22/50 (44.0%)	49/50 (98.0%)	18.44 ± 0.71	NR	7.71 ± 1.17
Athwal *et al*, 2017	LE + G (FSH) + HCG	65	28/65 (43.07%)	5/65 (17.85%)	4.3 ± 0.3	22.4 ± 0.5	9.62 ± 0.9	301.78 ± 85.7
	CC + G (FSH) + HCG	65	15/59 (25.42%)	2/59 (13.33%)	2.9 ± 0.7	22.7 ± 0.9	8.33 ± 0.4	464.7 ± 72.9
Chen *et al*, 2016	LE + HCG	52	NR	29/52 (55.7%)	2.2 ± 0.2	18.9 ± 1.3	11.7 ± 1.2	6052 ± 112.1
	LE	52	NR	16/52 (30.8%)	1.3 ± 0.3	19.3 ± 1.1	9.1 ± 0.2	319.3 ± 104.2
	CC	52	NR	17/52 (32.1%)	1.7 ± 0.5	19.0 ± 0.7	8.5 ± 0.5	327.7 ± 89.2
Foroozanfard *et al*, 2014	LE + HMG + HCG	50	NR	8 (20%)	1.86 ± 1.34	NR	7.52 ± 0.92	NR
	LE + HMG with LPS	50	NR	14 (35%)	1.78 ± 1.09	NR	7.76 ± 1.09	NR
	CC + HMG	50	NR	7 (17.5%)	1.78 ± 1.14	NR	7.73 ± 1	NR
	CC + HMG with LPS	48	NR	11 (27.5%)	1.78 ± 1.14	NR	7.62 ± 0.88	NR
Hosseini-Najarkolaei *et al*, 2020	LE + G	57	NR	Implantation rate 22%, clinical pregnancy rate 24 (42.1%)	NR	NR	9.45 ± 0.16	NR
	Artificial endometrial preparation	59		Implantation rate 20%, clinical pregnancy rate 22 (37.2%)			9.92 ± 0.19	
Harira *et al*, 2018	LE + HMG + HCG	106	91/106 (85.8%)	27/106 (25.4%)	1.66 ± 0.98	NR	9.8 ± 1.5	540 ± 180.5
	LE + CC	106	85/106 (80.2%)	32/106 (30.1%)	2.1 ± 1.01	NR	9.6 ± 1.7	650 ± 190.3

LE, letrozole; G, gonadotropins; CC, clomiphene citrate; NR, not reported; HMG, human gonadotropins; HCG, human chorionic gonadotropins; LPS, luteal phase support (mid-menstrual cycle).

### Pregnancy rates

**Figure 5.**
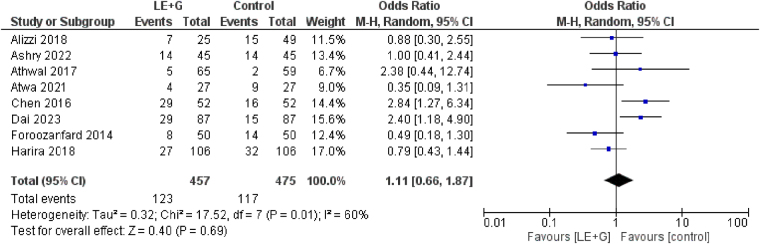
Forest plot comparing pregnancy rates between the treatment and control groups across the included studies.

We found no significant increase in the pregnancy rates in both groups (OR 1.11; 95% CI 0.66–1.87, *I*^2^ = 60%, *P* = 0.69). There was significant heterogeneity among studies (Fig. 5).

### Number of dominant follicles (n)

**Figure 6.**
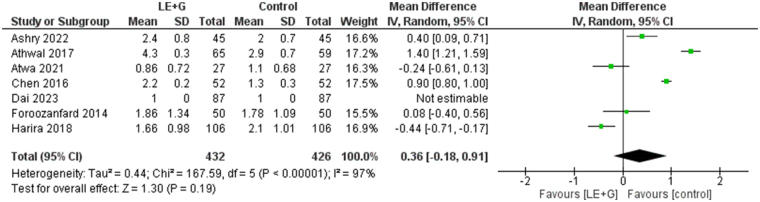
Forest plot comparing the number of dominant follicles between the sequential letrozole and gonadotropins therapy group and the control group.

We found no significant mean difference in the number of the dominant follicles (*n*) in both groups (MD 0.36; 95% CI −0.18 to 0.91, *I*^2^ = 97%, *P* = 0.19) The exclusion of the *Harira et al* (2018) in the sensitivity analysis resulted in a significant increase in the number of the dominant follicles in the control arm (MD 0.54; 95% CI 0.06–1.01). A significant heterogeneity was found among the studies (Fig. 6).

### Size of dominant follicles (mm)

**Figure 7.**
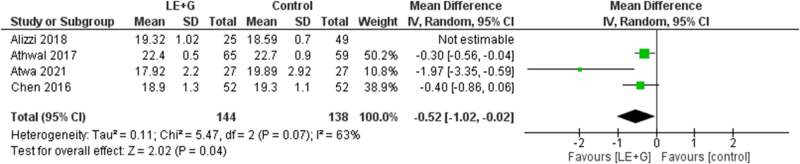
Forest plot comparing the dominant follicles (mm) size on day 14 of the menstrual cycle between the two groups.

There was a statistically significant reduction in the mean size of dominant follicles (mm) on day 14 of the menstrual cycle (MD −0.27; 95% CI −0.95 to 0.41; *I*^2^ = 63%; *P* = 0.04). There was significant interstudy heterogeneity (Fig. 7).

### Endometrial thickness (mm)

**Figure 8.**
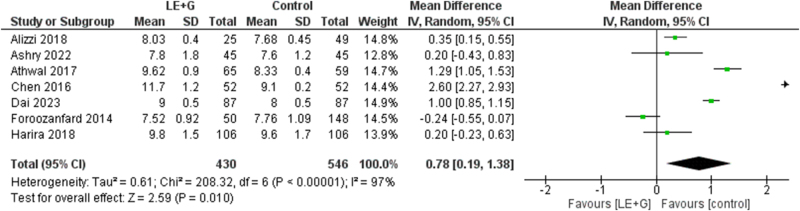
Forest plot comparing endometrial thickness in the sequential letrozole and gonadotropins therapy and control groups.

The comparative analysis showed a significant increase in the endometrial thickness (mm) in the treatment group (MD 0.78; 95% CI 0.19–1.38; *I*^2^ = 97%; *P* = 0.010). A significant heterogeneity was found among the studies (Fig. 8).

### Serum E2 levels (pg/ml)

**Figure 9.**
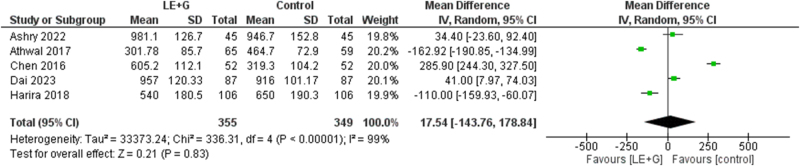
Forest plot comparing serum estradiol (E2) levels between the treatment and control groups.

There was no difference in the two groups for serum E2 levels (pg/ml) (MD 17.54; 95% CI −143.76 to 178.84; *I*^2^ = 99%, *P* = 0.83). There was significant interstudy heterogeneity (Fig. 9).

## Discussion

This meta-analysis included eight RCTs to evaluate the effect of LE + G therapy in women with PCOS compared to a range of standard ovulation induction protocols, including monotherapies, combination regimens, and adjunctive hormonal treatments.

The studies included in this meta-analysis enrolled women aged 18–40 years diagnosed with PCOS based on the Rotterdam Criteria. Participants had no endocrine abnormalities, and male partners had normal semen parameters. All had a history of infertility and received LE + G therapy. Only RCTs reporting outcomes such as ovulation, pregnancy rate, follicular development, and serum estradiol levels were included.

The LE + G group showed improved ovulation rates, although with moderate variation between studies. Sensitivity analysis revealed that removing one study shifted the benefit to the control group, suggesting that procedural factors like transvaginal needle ovarian puncture may influence outcomes. The treatment group also demonstrated increased endometrial thickness, suggesting improved endometrial receptivity. A small but significant reduction in the size of dominant follicles was observed, possibly reflecting more synchronized follicular development.

However, no significant difference was found in pregnancy rates between groups, indicating a disconnect between ovulation and conception. There was no major difference in the number of dominant follicles; when one study was excluded, the control group showed a significant benefit, indicating sensitivity to study design. Estradiol levels were similar across both arms, indicating a comparable hormonal profile. In summary, while sequential LE + G therapy may enhance ovulation and endometrial thickness in women with PCOS, it does not appear to improve pregnancy outcomes. These findings underscore the importance of standardizing treatment protocols and call for further high-quality studies, especially to evaluate the role of adjunct procedures like transvaginal ovarian puncture before ovulation induction.

In addition to the comparative analyses presented, understanding the clinical advantages of LE + G therapy is essential in managing ovulation induction in PCOS. Letrozole, an aromatase inhibitor, lowers E2 levels, creating a favorable hormonal environment for follicular development, reducing estrogen-related side effects and the risk of multiple pregnancies^[11]^. It is suitable as first-line or second-line therapy in CC-resistant cases without other fertility factors^[12]^. Letrozole may reduce ovarian hyperstimulation syndrome (OHSS) risk by lowering VEGF levels^[13]^ and enhancing ovarian response to gonadotropins^[14]^. Sequential therapy like LE + G improves ovulation while potentially reducing OHSS risk linked to gonadotropins.

This study is novel in its comprehensive evaluation of treatment outcomes beyond ovulation and pregnancy rates, including follicle development, endometrial thickness, and serum E2 levels, as well as a huge population pool due to the inclusion of 8 RCTs. In another systematic review and meta-analysis, Baradwan *et al*^[15]^, compared letrozole alone to LE + G therapy and favored LE + G, showing significant improvements in clinical pregnancy rates and mature follicle count. This is contradictory to our findings, where significant differences were not observed in favor of LE + G for the same outcomes. However, a key point of difference is that while our intervention arm was consistent with that of Baradwan *et al*, our control arm included different treatment approaches, as outlined in Table 1. Additionally, our analysis incorporated a larger patient pool, comprising 956 participants across 8 RCTs, compared to 723 participants across 6 RCTs in their study. Furthermore, we evaluated comparative outcomes not previously addressed, such as the size of dominant follicles and serum estradiol (E2) levels. These contradictory results may stem from differences in study design, sample sizes, and the variability in control interventions, which could impact treatment effectiveness and clinical outcomes. This highlights the importance of considering the broader context of treatment comparisons and emphasizes the need for further research to reconcile these differences.

During the literature search, we found that most of the articles focused on the comparison of L therapy alone (standard treatment) with various controls like CC alone, CC + Metformin, etc.^[3,11,12]^. These reviews favored L therapy, linking it to higher rates of ovulation and pregnancy. However, our review not only evaluated the efficacy of sequential LE + G therapy for women unresponsive to oral L but also encompasses a broader range of outcome measures, including ovulation rates, pregnancy rates, follicle development, endometrial thickness, and serum estradiol levels, offering a comprehensive view of treatment effects beyond just pregnancy outcomes and shedding light on various aspects of ovulation induction therapy in PCOS. Moreover, it evaluates a specific treatment regimen, discussing its efficacy against different interventions and offering focused insights into the effectiveness of LE + G therapy.

This meta-analysis provides insights into LE + G therapy for ovulation induction, increased endometrial thickness, but notable limitations must be considered. Among the eight included RCTs, only two had a high risk of bias, yet significant heterogeneity in outcomes. The heterogeneity observed in this meta-analysis arises from multiple factors across the included RCTs. First, Letrozole dosing varied: Chen *et al* used 2.5 mg, while Atwa *et al*, Ashry *et al*, and Harira *et al* used 5 mg. Second, Gonadotropin type also differed: Atwa *et al*, Ashry *et al*, Hosseini *et al*, and Foozonford *et al* used HMG, while Dai *et al* used recombinant FSH. Control arms included letrozole monotherapy (Ashry *et al*), CC combined with gonadotropins (Athwa *et al*), clomiphene or letrozole with luteal phase support (Foroozanfard *et al*)^[13]^, and artificial endometrial preparation protocols (Hosseini-Najarkolaei *et al*). Study populations differed: Atwa *et al* enrolled clomiphene-resistant women, while Dai *et al* included both insulin-resistant and non-resistant patients and conducted subgroup analysis based on insulin resistance and BMI. Most studies applied the Rotterdam criteria, but phenotype distribution was unreported. The number of treatment cycles analyzed also varied (one vs. up to three). Outcome definitions differed: Foozonford *et al* used serum progesterone ≥3 ng/ml to define ovulation, while Chen *et al* used ultrasound-confirmed follicle rupture. These differences in intervention, comparators, population, and outcome definitions contributed to moderate-to-high heterogeneity in pooled estimates^[14,16,17]^.

One of the key limitations of our systematic review and meta-analysis is its inability to stratify treatment effects by the various phenotypes of PCOS, commonly classified as phenotypes A, B, C, and D^[1]^. This limitation may hinder a more nuanced understanding of the comparative efficacy of L versus LE + G therapy in different PCOS subgroups. The significance of considering phenotypic variations lies in the fact that each phenotype is associated with distinct symptoms, pathophysiology, and risk profiles, which may influence treatment response. Studies as those by Vos *et al*^[18]^, *Alviggi et al*^[19]^, Pour *et al*^[20]^, and Patel *et al*^[21]^ have highlighted the variability in treatment outcomes depending on the PCOS phenotype, further emphasizing the need for individualized therapeutic approaches. The under-reporting of PCOS phenotypes poses a challenge to discerning the most appropriate treatment strategy for specific subgroups of women with PCOS. This limitation restricts the ability to develop personalized treatment plans, potentially leading to suboptimal outcomes in patients with varying phenotypic characteristics.

An important limitation of this meta-analysis is the inclusion of different control treatments across studies, which reduces the internal validity of the analysis by making direct comparisons less reliable. Since each control has different effects on ovulation and fertility outcomes, combining them may confound the results and contribute to the moderate-to-high heterogeneity (*I*^2^) observed in outcomes. As such, the pooled estimates reflect an average across different comparators and should be interpreted with caution.

Another significant limitation is the overall certainty of evidence ranged from moderate to very low, with ovulation supported by moderate-certainty evidence, while other outcomes suffered from low certainty due to methodological flaws and heterogeneity.

Future research should focus on large-scale, multicenter trials with standardized protocols to improve generalizability and minimize variability. Ensuring consistent comparator arms across studies would improve the validity of pooled estimates and allow for more reliable comparisons of treatment efficacy. Subgroup analyses based on PCOS phenotypes are important for future research, as different phenotypes may respond differently to treatment. Without this stratification, important differences may be missed, and personalized treatment strategies cannot be developed. Additionally, evaluating the long-term effects of LE + G therapy on pregnancy outcomes and potential adverse effects would provide valuable insights for clinical decision-making and patient counseling in the management of infertility in women with PCOS.

## Conclusions

This meta-analysis compared the effectiveness of sequential LE + G therapy for ovulation induction with traditional standard treatment options. The comparative analysis showed a significant increase in ovulation rates in the treatment group (OR 1.74; 95% CI 1.02–2.99; *P* = 0.04; *I*^2^ = 61%). However, when a sensitivity analysis was performed to address heterogeneity, the exclusion of one study (Atwa *et al*, 2021), which involved transvaginal needle ovarian puncture prior to ovulation induction, resulted in significantly higher ovulation rates in the control group (OR 2.02; 95% CI 1.38–2.95; *P* = 0.0003; *I*^2^ = 27%). Pregnancy rates were similar between groups. While the number and size of follicles on day 14 weren’t significantly different, trends suggested LE + G might have fewer and smaller follicles. LE + G was also linked to a thicker endometrial lining. These findings, along with the high variability among studies, highlight the need for further trials to assess the efficacy of LE + G in PCOS, particularly in protocols involving transvaginal needle ovarian puncture before ovulation induction.

## Data Availability

The data generated and analyzed during this study are available upon reasonable request. Data from the individual studies included in this meta-analysis can be found in the respective published articles.

## Research registration unique identifying number (UIN)

This systematic review and meta-analysis was prospectively registered in the International Prospective Register of Systematic Reviews (PROSPERO) with a protocol registration (CRD42023463333).
